# Pain medication use after spine surgery: is it assessed in the literature? A systematic review, January 2000–December 2009

**DOI:** 10.1186/s13104-015-1287-5

**Published:** 2015-07-29

**Authors:** Hiroyuki Yoshihara

**Affiliations:** Department of Orthopaedic Surgery and Rehabilitation Medicine, SUNY Downstate Medical Center, 450 Clarkson Ave, Brooklyn, NY 11203 USA; Department of Orthopaedic Surgery, Nassau University Medical Center, East Meadow, NY USA; Department of Orthopaedic Surgery, Nagoya City University Graduate School of Medical Sciences, Nagoya, Aichi Japan

**Keywords:** Pain medication, Spine surgery, Clinical outcome, VAS, Outcome measure, Narcotic

## Abstract

**Background:**

Spine surgery is one of the most difficult areas in which to achieve a good clinical outcome and pain medication is often used for a long period of time after surgery. The purpose of this study was to investigate whether pain medication use after spine surgery has been assessed previously with respect to clinical outcome.

**Methods:**

A systematic review of PubMed/MEDLINE databases was conducted from Jan 1st 2000 to Dec 31st 2009 using the search key words, “spine surgery” and “clinical outcome.” All publications reporting clinical outcomes were examined and analyzed for outcome measures and data with respect to pain medication use after spine surgery.

**Results:**

In total 990 articles met the inclusion criteria. Among them, 56 articles (5.7%) described definitive pain medication use after spine surgery; 98 articles (9.9%) used clinical outcome measures that incorporate pain medication assessment, although only one such study included a definitive description of pain medication use.

**Conclusions:**

Pain medication use after spine surgery was assessed in 15.5% of articles published during the last decade. The use of pain medication following spine surgery can affect clinical outcome and, therefore, needs to be taken into consideration for clinical assessment. In future studies, a detailed description of pain medication use and/or clinical outcome measures that incorporate pain medication assessment are advocated when reporting clinical outcomes after spine surgery so that it can be better assessed.

**Electronic supplementary material:**

The online version of this article (doi:10.1186/s13104-015-1287-5) contains supplementary material, which is available to authorized users.

## Background

Spine surgery is one of the most difficult areas in which to achieve a good clinical outcome after surgery. When compared to the other orthopaedic subspecialties, such as joint replacement surgery, a higher percentage of post-operative patients continue to have symptoms that require pain medication utilization and some require further surgeries. Failure rates associated with lumbar fusion surgeries range from 5 to 30% [1–10]. This high rate of unsuccessful spine surgery has generated the term ‘failed back surgery syndrome’.

The use of post-operative pain medication following spine surgery is nearly universal. However, despite the prevalence of use, there is no standardized protocol detailing the type, strength, or duration of pain medication use following specific procedures. Optimally, surgery should relieve the symptoms that brought the patient to surgery, with the termination of pain medication following the acute post-operative period, generally within one month. However, pain medication use is often continued long after this recovery period. The reason for pain medication implementation depends on individual circumstances; however, they are often needed for incomplete resolution of the symptoms for which surgery was originally indicated.

The frequency of spinal surgery is increasing worldwide, accompanied by an increasing number of publications that describe the clinical outcomes. It is controversial whether patients who continue to take pain medication, especially narcotic drugs, following spine surgery should be declared a surgical success. This can be misleading, even if the patient’s visual analogue scale (VAS) score, and/or clinical outcome measure shows improvement. Patients who continue to require strong pain medication after spine surgery may have been misdiagnosed pre-operatively and/or have previously undiagnosed additional spinal pathology. This article reviews previous descriptions or assessments of pain medication use after spine surgery in the literature when clinical outcomes were reported.

## Methods

A literature search was conducted of the PubMed/MEDLINE databases using the terms “spine surgery” and “clinical outcome” as search keywords. Then, the searching limits specified articles published from January 1st 2000 to December 31st 2009. Studies in the English language were included. A study was included if it reported the clinical symptom outcome of spine surgery with a minimum follow-up of 6 months. Editorials, review articles, basic science studies, case reports, and letters to the editor were excluded. A study was also excluded if it reported the clinical outcome of fractures, infections, or tumors or percutaneous procedures, and if the main patient population was younger than 20 years old.

Titles and abstracts of the identified studies were reviewed, and possible studies were retrieved in full text version. Selected manuscripts were analyzed and recorded according to the country of origin, type of study (prospective or retrospective), area of spine (cervical, thoracic or lumbar), diagnosis, surgical procedure, clinical outcome measures, follow-up period, and description of pain medication use before and after spine surgery.

## Results

**Fig. 1:**
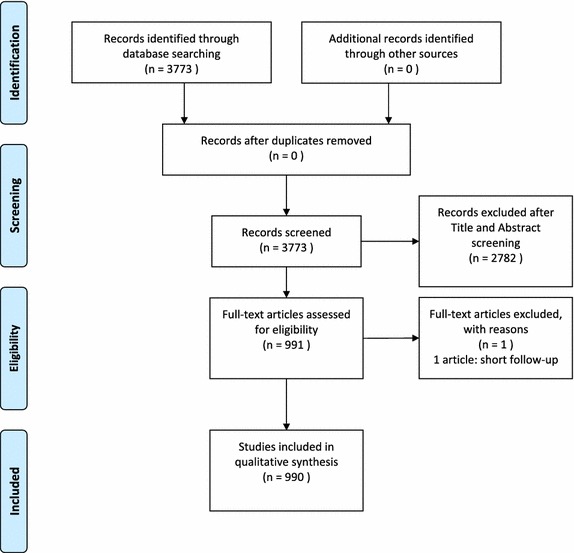
PRISMA flow diagram.

3,773 articles were identified form the literature search reporting on spine surgery and clinical outcome (Fig. 1). A total 991 articles were identified in the initial query from the title and abstract. All of these 991 articles were read and analyzed with the full-text review. One article was excluded due to short follow-up period. A total of 990 articles were finally included in this study.

Fifty-six articles (5.7%) described pain medication use after spine surgery in detail (Table 1, Additional file 1: Table S1). Among the 56 articles, 33 articles (3.3%) described pain medication use before and after surgery in detail (Table 1, Additional file 1: Table S1). 98 articles (9.9%) used clinical outcome measures incorporating a pain medication use assessment (Table 1). One article included both a description of pain medication use after surgery and a clinical outcome measure that incorporated pain medication use assessment. Therefore, pain medication use after spine surgery was assessed in 153 articles (15.5%). Details of the clinical outcome measures of 98 articles were described as follows: 36 articles used the SRS-Questionnaire, 10 articles used the Prolo scale, 6 articles used the Modified Stauffer-Coventry score, 5 articles used the Robinson scale, 5 articles used the Beaujon score, and 37 articles used other clinical outcome measures (Table 2, Additional file 2: Table S2). One article used both SRS-Questionnaire and Prolo scale.

**Table 1:** Summary of the study result

	No. of articles
Articles met the inclusion criteria	990
Pain medication use description after surgery	56 (5.7%)
Pain medication use description before and after surgery	33 (3.3%)
Clinical assessment measure which incorporate pain medication use	98 (9.9%)

One article included both description and clinical assessment measure.

**Table 2:** Details of clinical assessment measure which incorporate pain medication use (among 98 articles)

	No. of articles
SRS-Questionnaire	36
Prolo scale	10
Modified Stauffer-Coventry score	6
Robinson scale	5
Beaujon score	5
Other clinical outcome measures	37

One article used both SRS-Questionnaire and Prolo scale. Many of other clinical outcome measures were authors’ own scale.

## Discussion

Physicians can assess functional impairment objectively; however, pain is generally only measurable by the patient in a subjective assessment. Due to this subjective nature, pain is difficult to assess clinically. Strong indications for spine surgery are instability of the spine and neurological deficits including motor deficit and bladder and bowel dysfunction, while pain remains strictly a relative indication. However, an increasing number of spine surgeries are recently being performed based mainly on pain symptoms.

In the clinical setting, it is not uncommon for spine surgeons to encounter patients who continue to use pain medications up to 1 year following spine surgery. This is consistent with the present literature review, with some articles describing continuous pain medication use by a significant proportion of patients beyond 1 year after spine surgery. Ali et al. [11] reported that 35% of patients continued to take pain medications for symptoms at 38 months following fusion surgery for adult idiopathic scoliosis. In comparing lumbar disc arthroplasty to fusion for single-level degenerative disc disease, Blumenthal et al. [12] reported a narcotic use rate of 64% in the arthroplasty group and 80.4% in the lumbar fusion cohort at 24 months after surgery. Despite this high narcotic requirement, these patients were within a subgroup of patients that were categorized as a clinical success. Hallet et al. [13] reported that 64–83% of patients with lumbar single-level disc disease were taking at least one oral strong analgesic or anti-inflammatory agent at 2 years after decompression or fusion surgery. Among patients reported as achieving overall success following surgery for single-level lumbar degenerative disc disease at 24 months, Zigler et al. [14] reported that 31% of ProDisc patients and 39% of fusion patients remained on narcotics. While some patients may develop additional spine pathology 5–10 years after surgery, these conditions should not alter the 1–2 year outcomes after surgery. Thus the surgery may not necessarily be as successful as the outcome measures would indicate in patients showing an improved VAS score or clinical outcome measures after surgery, who nevertheless continue to take strong pain medications. Indeed, some other pathologies of the spine might remain or the surgery may not have been indicated for the pathology diagnosed. In fact, Epstein et al. [15] reported that 47 (17.2%) of 274 spinal consultations seen by a single neurosurgeon were scheduled for “unnecessary surgery”.

Pain medication regimens for spine diseases may differ among countries. In the author’s experience, pain medication regimens are quite different between Japan and the United States. In Japan patients with spinal pathologies rarely take narcotic pain medications before or after surgery. In contrast, in the US, it is a very common practice. This difference of clinical practices is likely multifactorial. In the US, the expectation of patients undergoing surgery is one of no or minimal pain. Furthermore, it is the expectation of the surgeon that the patient be discharged from hospital often within a few days after surgery, necessitating the use of strong pain medications in the early postoperative period. However, some patients continue to take narcotic pain medication months or even years after surgery. In contrast, few patients in Japan are prescribed narcotic pain medication after spine surgery; instead they receive oral or suppository non-steroidal anti-inflammatory drugs (NSAIDs). Patients also tend to remain in the hospital following spine surgery for 7–14 days in Japan through the acute postoperative period, often resulting in much less pain upon discharge. Clinical outcome assessment is usually performed 6 months or 1 year post-surgery, so short-term pain medication use does not have an impact on such assessment; however, if the patient is still taking pain medication at the assessment period, it can have a considerable effect on these outcome measures.

There are numerous reports of clinical outcome assessments following spine surgery in the literature, reporting a variety of clinical outcome measures. The most frequently used are the VAS score, Oswestry disability index (ODI), SF-36, Roland-Morris Questionnaire, Japanese Orthopaedic Association (JOA) score, Scoliosis Research Society (SRS)-Questionnaire, Odom score, Nurick scale, and neck disability index (NDI). However, most of these measures do not include an assessment of pain medication use. Some clinical outcome measures do incorporate such assessments, namely the SRS-Questionnaire, Prolo scale, Stauffer-Coventry score, Robinson scale, and Beaujon score. Indeed, all of these measures have a separate component for pain medication use. In the SRS-Questionnaire, pain medication use is a variable within the pain related section. In addition to the clinical outcome measures listed above, many clinical studies use the VAS score alone to assess pain. Thalgott et al. [16] reported that following circumferential anterior lumbar interbody fusion, the average back pain result for ‘with medication’ was 3.21 (scale of 1–10), and for ‘without medication’ was 5.58. Therefore, there was a 2.37 points difference in the score depending on medication use. Consequently, pain medication usage is a very important factor to consider when accurately assessing pain scores and clinical outcomes following spine surgery and, therefore, preoperative as well as postoperative pain medication usage needs to be considered in order to make a valid assessment of postoperative clinical outcomes and/or pain. The type, dosage, and frequency of pain medications may also need to be addressed. For example, narcotics usage likely effects the pain assessment more than NSAIDs use. Furthermore, future studies are expected to assess the effect of each pain medication relative to the VAS score, and modify the score accordingly. Alternatively, a new pain assessment measure may need to be devised that validly incorporates pain medication utilization.

## Conclusions

Pain medication use after spine surgery was assessed in 15.5% of articles published over the past 10 years. Pain medication can affect clinical outcomes. Therefore, a detailed description of pain medication use and/or clinical outcome measures that incorporate pain medication assessment are advocated when reporting clinical outcomes after spine surgery in the literature so that it can be better assessed; otherwise, the outcomes may be misleading.

## Additional files

Additional file 1:
**Table S1.** Articles that described pain medication use after spine surgery in detail. Additional file 2:
**Table S2.** Clinical outcome measures that incorporate pain medication use.
