# Supplementary material for: Pain medication use after spine surgery: is it assessed in the literature? A systematic review, January 2000–December 2009
Source: BMC Res Notes. 2015 Jul 29;8:323. doi: 10.1186/s13104-015-1287-5 (PMC4518636; doi:10.1186/s13104-015-1287-5)
Supplement: Additional file 1: — Table S1. Articles that described pain medication use after spine surgery in detail. [file 13104_2015_1287_MOESM1_ESM.pdf]

Additional Table 1. Articles that described pain medication use after spine surgery in detail

Authors & Year	Country	Type of study	C, T, L	Diagnosis	Surgical procedure	Clinical outcome measure	Mean follow-up period	Pain meds description bef surg.	Pain meds description after surg. (at follow-up)
Barrick, 2000	US	Retro	L	Painful discs within a solid posterolateral spinal fusion	ALIF	ODI, NRS, etc	58 months	+	Before surgery, 17 taking opioids, as comared with 13 taking them afterward. However, opioid use decreased in all.
Sampath, 2000	US	Pro	C	Myelopathy	Decompression and/or fusion	Satisfaction rating, pain rating, etc	11.2 months	+	Opiate use is increased from 16.7% at enrollment to 20.8%.
Freeman, 2000	UK	Retro	L	Lumbar disease	PLIF	Pain, patient satisfaction, etc	12 months	-	48 pts; none:30, occasional:16, regular:2
Edwards, 2000	US	Retro	C	Myelopathy	Laminoplasty	Nurick scale	24 months	+	39% pre-op required narcotics, no pts taking narcotics at latest follow up.
Heller, 2001	US	Pro	C	Myelopathy	Laminoplasty, laminectomy and fusion	Nurick grade	25.5 and 26.2 months	-	One patient in each group (13 patients each) was taking narcotic analgesics.
Grob, 2001	Switzerland	Retro	C	Degenerative disease	ACDF	VAS	34 months (22-46)	+	In the table. Pain medication use: 5/24 for plate group, 8/26 for non plate group
Kim, 2002	Korea	Pro	T, L	Ankylosing spondylitis	Fusion	Arthritis Impact Measurement Scales	36 months	-	Patients used less analgesic medications after surgery.
Gardner, 2002	UK	Retro	L	DDD	Graf band	ODI, Disability & Risk assessment method	7.4 years (5.6-8.5)	-	One-quarter said they never used analgesics, and 54% said they sometimes used them, with 13% using them more than daily.
Zelle, 2002	Germany	ND	L	Spondylolisthesis, DDD, etc	Circumferential fusion	Pain scale (0-10), etc	Minimum 12 months	-	About half of the patients were on regular use of analgesics.
Schofferman, 2002	US	Retro	C	Cervicogenic headache	ACDF	NRS, ODI	37.3 months	+	4 patients were no longer taking opioid analgesics; 3 still required opioids for residual neck pain; and 2 were taking opioids for other painful problems (total 9 pts).
Johnsson, 2002	Sweden	Pro	L	Spondylolytic spondylolisthesis	Non-instrumented posterolateral fusion	Pain	1 year	-	Regular need of analgesics: 2/10 in the OP-1 group, 3/10 in the autograft group
Martin-Benlloch, 2003	Spain	Retro	C	Spondylotic myelopathy	Laminoplasty	Nurick, Robinson pain scale	24 months (19-38)	-	One patient (1/12) required NSAIDs.
Ali, 2003	US	Retro	T, L	Adult idiopathic scoliosis	Fusion	Questionnaire	2 years	-	8/28 (35%) continued to take pain medication for symptoms.
Görgülü, 2004	Turkey	ND	L	Disc herniation	Discectomy	Hannover Questionnaire, VAS, etc	2.6 years	-	Regular use of analgesics was in 8.4% of fat graft group and 3.9% for w/o fat graft group

Kienapfel, 2004	Germany	Retro	C	Radiculopathy or myelopathy	ACDF	Cervical Spine Symptom Scale, etc	7 years (3-12)	+	In the table. Pain medication use: 10 patients (10/54)
Kim, 2005	Korea	Retro	L	Spondylolisthesis, foraminal stenosis	Fusion	VAS, JOA	20.6 and 21.5 months	+	1/11 in the open pedicle screw group needed oral nonopioid analgesic medication 1 year follow-up.
Potter, 2005	US	Retro	L	DDD, DLS, spondylolistehsis	TLIF	VAS, RMDQ, etc	34 months (24-61)	+	24 (29%) reported occasional or greater narcotic use for pain control. 58 (71%) were entirely narcotic-free.
Galiano, 2005	Austria	Retro	L	LSS	Laminectomy	VAS, ODI	2.7 years	+	63% of the patients did not take any analgesics at all, 21% did occasionally (less than once a day), 16% regularly.
Schwender, 2005	US	Retro	L	Disc herniation, spondylolisthesis	TLIF	VAS, ODI	22.6 months (18-28)	-	Narcotic use was discontinued, on average, between 2 and 4 weeks postoperatively.
Wenger, 2005	Switzerland	Retro	L	Isthmic spondyllisthesis	Posterior instrumentation and posterolateral fusion	VAS, questionnaire	9.9 years (0.5–19.4)	-	In 45.5% of the patients analgesic medications were not required, and 43.9% required them sporadically.
Bertagnoli, 2005	Germany	Retro	C	Radiculopathy	TDR	VAS, ODI	12.7 months (12-14)	+	In the table. Regular medication usage (%): NSAIDs (28.7), Narcotics (8.9), Tramadol (12.8)
Blumenthal. 2005	US	Pro	L	Single-level DDD from L4-S1	TDR, lumbar fusion	VAS, ODI, SF-36	24 months	+	In the subgroup of patients demonstrating clinical success, there was a significantly lower rate of narcotic usage for pain in the investigational group (64.0%, 73 of 114) compared with the control group (80.4%, 37 of 46).
Woiciechowsky, 2005	Germany	Retro	C	Spinal canal stenosis	Corpectomy	Odom	21-60 months	-	No patient needed permanent pain medication beyond the sixth postoperative week. However, 5 /20 patients used sporadic NSAIDs.
Mofidi, 2005	Ireland	Retro	L	Back pain with or without radiculopathy	PLIF	ODI, questionnaire	4.4 years (2-7)	+	43 patients (77%) did not need routine analgesia for back pain, 31 (55%) of whom no longer required analgesia for back-related symptoms.
Gelalis, 2006	Greece	ND	L	Degenerative lumbar spinal stenosis	Decompression with or without fusion	Questionnaire	11.6 years	-	29 patients did not use any analgesics, 10 used analgesics weekly, 7 used them almost every other day and 4 used them consistently.
Chiba, 2006	Japan	Retro	C	CSM, OPLL group	Laminoplasty	Axial pain, JOA	14.2 years (10-22)	-	Less than a third of post-op axial pain patients in both groups were taking daily antiinflammatory drugs to relieve such symptoms.
Swan, 2006	US	Pro	L	Isthmic spondlylolisthesis	Anterior and/or posterior fusion	VAS, ODI, etc	24 months	+	In the table. 7/47 in the combined AP fusion group and 11/46 in the posterolateral fusion group used narcotic medicine.
Carragee, 2006	US	Pro	L	Discogenic pain, unstable spondylolisthesis	Anterior and posterior fusion	VAS, ODI, etc	24 months	-	In the table. 3% (1/32) and 50% (15/30) used narcotic medicine for each group.

Chung, 2006	Korea	Pro	L	Back pain	TDR	VAS, ODI	37 months (25-42)	+	34 patients (34/36) reported no need for medication including NSAIDs or narcotic medication. One patient required pain medication occasionally, and another daily.
Schnake, 2006	Switzerland	Pro	L	LSS with degenerative spondylolisthesis	Dynamic stabilization	VAS, walking distance, Prolo Economic Scale	Minimum 2 years	+	6 (25%) used analgesics.
Bertagnoli, 2006	Germany	Pro	L	Discogenic pain	TDR	VAS, ODI, patient satisfaction score	34.6 months (24-56)	+	Regular medication usage (%): NSAIDs (15), Narcotics (0), Tramadol (0)
Bertagnoli, 2006	Germany	Pro	L	Adjacent segment disease s/p fusion	TDR	VAS, ODI, patient satisfaction score	27 months (24-48)	+	Regular medication usage (%): NSAIDs (20.0), Narcotics (0), Tramadol (42.8)
Carragee, 2006	US	Pro	L	Disc herniation	Discectomy	VAS, ODI	2 years	+	Limited discectomies/subtotal discectomies; daily non-narcotics: 7(15.2%)/6(20.0%), daily narcotics: 2(4.3%)/2(6.7%)
Bertagnoli, 2006	Germany	Pro	L	Discogenic pain	TDR	VAS, ODI, patient satisfaction score	Minimum 2 years	+	Regular narcotics use: 5% in the smoker group and 4% in the non-smoker group
Hwang, 2007	Taiwan	Retro	C	DDD	ACDF or corpectomy	VAS, JOA	24 months	-	Eight patients had chronic pain from donor site and needed analgesic drugs
Amit, 2007	UK	ND	C	Spondylosis	TDR	VAS, SF-36, NDI, Odom	12 months	-	All but one patient reported decreased or complete cessation of the requirements for pain killers.
Yukawa, 2007	Japan	Pro	C	Myelopathy	Laminoplasty or skipping laminectomy	VAS, JOA	mean 28.1 months	-	There were no patients with a daily demand of NSAIDs for axial neck pain.
Hallett, 2007	UK	Pro	L	Single level DDD	Decompression with or without instrumented fusion	VAS, SF-36, RMDQ	5 years	+	69%, 83%, and 64% of the patients in Groups 1 to 3, respectively, were taking at least 1 oral strong analgesic or anti-inflammatory agents at 2 years follow-up.
Zigler, 2007	US	Pro	L	DDD	TDR or circumferential fusion	VAS, ODI, SF-36	24 months	+	Of patients achieving overall success, only 31% of fusion and 39% of TDR patients remained on narcotics. In patients not achieving overall success, narcotic usage remained relatively unchanged (76% fusionl and 79% TDR).
Lind, 2007	Sweden	Pro	C	Radiculopathy	ACDF	VAS, Odom	2 years	-	20/22 were totally freee from analgesics and two reduced their intake of analgesics.
Peele, 2007	US	Retro	C	Radiculopathy	ACDF	Pain	1 year	-	No patients required prescription analgesics at their 2-week follow-up vist.
Mirzai, 2007	Turkey	Pro	L	Disc herniation	Nucleoplasty	VAS, ODI	12.1 months (10-15)	+	In the table. 46 (94%) stopped or reduced analgesics.
Lawrence, 2008	US	Retro	C	Radiculopathy	Cervical arthrodesis	Modified Robinson criteria	31-32 months	+	16 (34%) continued to require chronic narcotic pain medication up to 2 years after surgery.

Schaeren, 2008	Switzerland	Pro	L	LSS with degenerative spondylolisthesis	Decompression and dynamic stabilization	VAS, walking distance, etc	Minimum 2 years	+	14 patients (68%) did not use pain medication any more, 6 patients needed NSAIDs and no patient was in need of opiates.
Barth, 2008	US	Pro	L	Disc herniation	Microdiscectomy or microscopic sequestrectomy	VAS, SF-36	24 months	-	In the table and figure. About 10 and 20% of patients for each group took frequent to regular pain medicine.
Butterman, 2008	US	Pro	C	Degenerative disease	ACDF	VAS, ODI	2-3 years	+	In the table. 19% and 23% took narcotics at 1-2 year follow-up for each group.
Shabat, 2008	Israel	Pro	L	LSS	Decompression surgery	VAS, Barthel index, walking distance	mean 36.8 months	+	In the table. A significant decrease in analgesic drug consumption was noticed.
Willén, 2008	Sweden	ND	L	Lumbar spinal stenosis	Decompression with or without fusion	ODI, SF-36, VAS, etc	1 to 6 years	-	3 patients (12%) used analgesics routinely.
Falavigna, 2009	Brazil	Pro	C	Disc herniation, CSM, spondylosis	ACDF	Odom	31.8 ± 18.8 months	+	8 patients (20%) used analgesic (mean frequency use was 2.6 times weekly).
Crandall, 2009	US	Pro	L	DLS, LSS, spondylolisthesis	TLIF or ALIF	VAS, ODI	38 months (24-68)	+	The decline in the need for pain medication was nearly identical between groups, with a score of 2.62 preoperative improving to 1.94 at 1 year and 1.75 at latest (their own pain medication score).
Dimar, 2009	US	Pro	L	DDD	Posterolateral fusion with illiac graft	ODI, SF-36, leg and back pain	24 months	+	The proportion of patients taking weak narcotic medication is significantly less at 2 years compared with preoperative (51.8% vs. 31.7%). Also, the proportion of patients taking strong narcotic medication is significantly less at 2 years compared with preoperative (18.4% vs. 9.9%).
Ahrns, 2009	Germany	Pro	L	DDD	Lumbar nucleus replacement	VAS, ODI	24 months	+	In the figure. Analgesic or narcotic drug use was nearly nonexistent.
Beaurain, 2009	France	Pro	C	DDD	TDR	VAS, NDI	24 months	+	In the figure. Analgesic use also strongly decreased: before the surgery 89.4% (59/66) of the patients were under medical treatment for pain, versus 22.2% (14/63).
Thalgott, 2009	US	Pro	L	IDD, DDD, or herniated nucleus pulposus	Circumferential anterior interbody fusion	VAS, ODI, SF-36	24 months	+	The average 1 to 10 pain results for “back with medication” was 3.21, and the average change for ”back with medication” was 2.08. The average for “back without medication” was 5.58, and the average change for “back without medication” was 2.65.
Ruetten, 2009	Germany	Pro	L	Disc herniation	Micro or full endoscopic discectomy	VAS, ODI, etc	24 months	-	Postoperative pain medication were significantly reduced in the full endoscopic group.

Murrey, 2009	US	Pro	C	Disc disease	TDR	VAS, NDI, SF-36	24 months	+	13.0% (73% decrease) of Fusion patients and 11.2% (77% decrease) of TDR patients remained on weak or strong narcotics.

Pro = prospective; Retro = retrospective; C = cervical spine; T = thoracic spine; L = lumbar spine; DDD = disc degenerative disease; LSS = lumbar spinal stenosis; CSM = cervical spondylotic myelopathy; DLS = degenerative lumbar scoliosis; OPLL = ossification of posterior longitudinal ligament; IDD = internal disc disruption; ACDF = anterior cervical decompression and fusion; TDR = total disc replacement; ALIF = anterior lumbar interbody fusion; TLIF = transforaminal lumbar interbody fusion; ODI = Oswestry disability index; NRS = numerical rating scale; VAS = visual analogue scale; RMDQ = Roland Morris disability questionnaire; NDI = neck disability index; AP = anterior and posterior; NSAIDs = non-steroidal anti-inflammatory drugs; ND = not described
