# Supplementary material for: Pain medication use after spine surgery: is it assessed in the literature? A systematic review, January 2000–December 2009
Source: BMC Res Notes. 2015 Jul 29;8:323. doi: 10.1186/s13104-015-1287-5 (PMC4518636; doi:10.1186/s13104-015-1287-5)
Supplement: Additional file 2: — Table S2. Clinical outcome measures that incorporate pain medication use. [file 13104_2015_1287_MOESM2_ESM.pdf]

Additional Table 2. Clinical outcome measures that incorporate pain medication use

SRS-Questionnaire (pain medication related questionnaire)

Which one of the following best describes your medication usage for your back?	
Score	
5	None
4	Non-narcotics weekly or less (e.g., Aspirin, Tylenol, Ibuprofen)
3	Non-narcotics daily
2	Narcotics weekly or less (e.g., Tylenol #3, Lorocet, Percocet, Darvocet)
1	Narcotics daily

Prolo scale

Grade	Pain	Function	Economic	Medication/Day
1	excruciating or unbearable	total incapacity	unable to do tasks around the home	≥10 hydrocodone tablets or equivalent
2	severe	able to do activities in the home	able to do tasks around the home but unable to work	6-9 hydrocodone tablets or equivalent
3	moderate	able to do activities outside the home w/ limitation of moderate-demand activities	able to work at moderate capacity	3-5 hydrocodone tablets or equivalent
4	mild	limitation of strenuous activities or sports	able to work at moderate capacity	regular NSAIDs &/or occasional hydrocodone tablets
5	no pain	able to do all activities	able to work at heavy capacity or previous occupation	no or occasional NSAIDs or equivalent

* A total score is that derived from the sum of pain, function, economic, and medication grades: poor, 4 to 8; fair, 9 to 12; good, 13 to 16; and excellent, 17 to 20. Abbreviation: NSAIDs = non-steroidal anti-inflammatory drugs.

Modified Stauffer-Coventry score

	Grade					
Parameter	0	1	2	3	4	Total
Low back pain	Permanent	Frequent	Moderate	None		3
Radicular pain	Permanent	Frequent	Effort	None		3
Neurologic deficit	Major		Moderate		None	4
Medication	Major	Moderate	None			2
Day living activities	Impossible		Normal			2
Work status postoperatively	No work	Frequently stopped	Change	Same work >6 mos. Change <3 mos.	Normal	4
Psychiatric status	Preoperative	Secondary to pathology	None			2
Total						20

Robinson scale

Outcome	Pain	Medication	Activity
Excellent	None	None	Normal
Good	Mild	Occasional anti-inflammatories	Normal
Fair	Moderate	Frequent anti-inflammatories	Restricted
Poor	Severe	Narcotics	Incapacitated

Beaujon score

Signs and Symptoms	Score
Neurogenic claudication	0 Only able to walk <100 m
	1 Able to walk 100–500 m
	2 Can walk >500 m
	3 No restriction in walking
Leg pain at rest	0 Continuous severe pain
	1 Occasional severe pain
	2 Occasional mild pain
	3 None
Leg pain at exertion	0 Severe, immediately after starting to exercise
	1 Episodic with late onset
	2 None
Low back pain	0 Continuous severe pain
	1 Occasional severe pain
	2 Occasional mild pain
	3 None
Neurological deficit (motor or sensory)	0 Major (grade 0–3) or sphincter dysfunction
	2 Slight (grade 4)
	4 None
Medications required	0 Major analgesics
	1 Occasional use of minor analgesics
	2 None
Quality of life	0 Completely incapacitated
	1 Very limited
	2 Slightly limited
	3 Normal
Maximum score	20
